# Psychiatric Comorbidity in Patients from the Addictive Disorders Assistance Units of Galicia: The COPSIAD Study

**DOI:** 10.1371/journal.pone.0066451

**Published:** 2013-06-18

**Authors:** César Pereiro, Carlos Pino, Gerardo Flórez, Manuel Arrojo, Elisardo Becoña, COPSIAD Group

**Affiliations:** 1 Drug Dependency Unit, A Coruña, Spain; 2 Drug Dependency Unit, Pontevedra, Spain; 3 Addictive Behavior Unit, Ourense, Spain; 4 Mental Health and Drug Dependency Assistance Service, Direction of Sanitary Assistance, Galician Health Service, Galicia, Spain; 5 Faculty of Psychology, University of Santiago de Compostela, Santiago de Compostela, Spain; University of South Florida, United States of America

## Abstract

The objective of this study is to assess the prevalence of psychiatric comorbidity in patients under treatment within the addictive disorders assistance units of Galicia (Spain).

**Material and Methods:**

A total of 64 healthcare professionals performed clinical diagnosis of mental disorders (on DSM IV-TR criteria) in 2300 patients treated throughout March 2010 in 21 addictive disorders assistance units.

**Results:**

56.3% of patients with substance abuse/dependency also showed some other mental disorder, 42.2% of patients suffering from at least an Axis I condition and 20.2% from some Axis II condition. Mood and anxiety disorders and borderline and antisocial personality disorders were the most frequent disorders in both axes.

**Conclusions:**

A high comorbidity was found between mental and substance use disorders (SUD) in patients seen at the addictive disorders assistance units of Galicia.

## Introduction

Dual disorder is usually the term of choice used in describing the co-occurrence of a substance use disorder (SUD) and another mental disorder [1], [2]. Although this association has been known for 30 years, it was only in the last two decades that it has increasingly become a source of interest largely because high prevalence has been reported in the literature and the negative influence such comorbidity may have on the evolution and prognosis of both disorders [3].

This high prevalence found since the 1990s has been associated to: the decrease in the number of long-term inpatients in psychiatric hospitals where there was less access to toxic substances; greater access (in both availability and price) to substances of abuse (and particularly stimulants), improvement of drug addiction assistance networks which has resulted in better catchment and care of patients (and therefore of registered cases) and an increase in diagnosis as professionals have a greater understanding of this problem [3].

A number of studies report a high comorbidity between SUD and mental disorders [4]–[8], with a highly variable prevalence as studies have been conducted in heterogeneous populations (clinical, prison, general populations) and using different methodologies.

Regier *et al.* (1990) [4] reported that 44% of alcohol abusers and 64.4% of illegal substance abusers that had started treatment for drug use showed at least a major mental disorder while Kessler *et al*. [5], [6] found in a study of general population that persons diagnosed with alcohol dependence were 4.1 times more likely to suffer a mental disorder than non-alcohol dependent population and that among persons with illegal drug dependency the risk was 4.9 times higher.

The prevalence of dual disorder is noticeably higher in treatment samples than in community samples [9], as the latter are not confined to persons requesting professional assistance [4], [10].

A particularly interesting study of clinical populations was conducted in Canada by Rush & Koegl (2008) [11]. It comprised 9839 patients from different health programs from the system of mental health services of the Ontario area and it found a global prevalence for dual disorders of 18.5%, which could be more representative of clinical reality.

The following variables have been associated with an increased risk of suffering a dual disorder: male gender, young age, low education, single, urban milieu and better premorbid level of functioning, family history of substance abuse and a history of traumatic life events [12].

Alcohol is the most frequent substance of abuse among dual patients, followed by cannabis or cocaine, although the use of amphetamines, opiates, hallucinogens, sedative agents, nicotine as well as caffeine is also frequent. No psychopathology-related preference has been reported, being substance availability the determining factor for use [12].

As to evolution and prognosis, patients with dual disorders have a greater suicidal risk [11], [13], greater use of ER visits and psychiatric hospitalization as consequence of both a worsening of symptoms as in the case of alcohol use in patients with affective disorders and decreased treatment compliance [14], [15], a worse response to treatment with increased side effects and interactions [16], a greater occurrence of violent behavior [17], [18], greater family instability and social exclusion [16], [19], [20], a greater victimization risk [21], an increased risk of legal problems [12], [17], and a greater prevalence of communicable diseases associated to risk-taking behaviors [22].

It should be borne in mind that among patients with major psychiatric disorders and co-occurring substance abuse-related problems, the prevalence of impulsive and antisocial behavior is higher than among non-substance abuse psychiatric patients. These behaviors accelerate the onset of both disorders, lead to the use of multiple substances and therefore, result in increased problems, more serious legal repercussions and decreased adaptation to the social, family and occupational environment [23].

Therapeutically, patients with dual diagnosis are both a challenge and a dilemma for a healthcare system traditionally organized into two parallel assistance services, one for mental health and one dealing with addictions. Each healthcare service tends to give treatment priority to the disorder that has traditionally been within its scope while ignoring the other disorder, thus making patient’s recuperation and improvement less likely [3].

On the other hand, the effectiveness of existing programs and interventions to treat dual disorders in a specific and comprehensive manner has not been clearly proven [24]. Generally, the different interventions may be applied in dual disorders. In other words, the treatments that reduce psychiatric symptomatology also work in dual patients and the same is true of treatments that reduce substance abuse [25].

Another important methodological issue in our study is data collection on toxic substance use, a greatly discussed factor in the literature on dual disorders. Since studies indicate that over 50% of patients are polydrug users, it does not seem viable to confine studies to patients using only one toxic substance [26].

Indeed, Rounsaville *et al.* (2003) [27] suggested that a detailed study should be conducted on the use of the different toxic substances of abuse found in the environment so as to determine which one was the main substance of abuse and consequently focus attention on that substance. However, the determination of which the main substance of abuse is has proved to be heterogeneous as in some studies this term refers to the most used substance while in others it refers to the substance that leads to the greatest demand of treatment and finally others use the term to speak of the substance that generates the greatest disability [26].

The use of structured and semi-structured interviews or questionnaires to be filled out by patients and their relatives also results in significant differences when measuring the main substance of abuse [26].

Consequently, it is hardly surprising that dual disorders represent a challenge not just for clinicians but also for healthcare managers. In this regard, the main objective of this study was to determine the prevalence of dual disorders in the addictive disorders assistance units of Galicia (Spain).

## Materials and Methods

This is a cross-sectional, descriptive and naturalistic study of mental and substance use disorders (SUD) in patients seen at the additive disorder assistance units of Galicia in the northwestern region of Spain with a population of 2,797,653. The addictive disorders assistance service comprises 17 drug dependency units and 6 alcoholism units, where 84 healthcare professionals work (51 psychologists and 33 doctors).

Our definition of main substance was a substance that met the following conditions: the most frequently used, the one leading to treatment demand and greatest disability.

### Sample

The study was conducted with all patients seen between the first and the thirty first day of March 2010. This cross-sectional strategy in a clinical population in which a sufficient period of previous treatment and assessment is ensured has already been used in other studies [11].

Protocols from 2560 patients were received, of which 260 (10.15%) were excluded as they did not comply with eligibility criteria or were incomplete.

The final sample consisted of 2300 patients, 1834 (79.7%) were male and 466 (20.3%) female. The mean age was 41.27 (SD: 10.13) (range: 18–64 years). All of them were being treated at the addictive disorders assistance units of Galicia as they had problems of abuse or dependence on one or several psychoactive substances.

### Procedure

An *ad hoc* data collection protocol was written that included sociodemographic variables, substances used (main and others) and diagnosis of mental and use of substances disorders (DSM IV-TR criteria) (APA, 2000*)* [28].

Protocols were filled out by 64 out of the 84 (76%) healthcare professionals of the addictive disorders assistance units of Galicia.

Eligibility criteria were the capability to understand and sign informed consent, age between 18 and 65 years and having been treated for at least three months in the drug dependence or alcoholism unit so that diagnoses were always based on a longitudinal assessment made by expert professionals and on the basis of all data available using the LEAD (longitudinal expert with all data) method [20].

As it is frequent to find acute psychiatric symptomatology in users of psychoactive substances without this implying that there is basis for diagnosing a disorder, symptom persistence is necessary for confirmation of that diagnosis [28].

Proper consideration must also be given to the presence of behavioral changes in substance users from the states of intoxication and abstinence which may be confused with personality disorders. In this case persistence of alterations over the time is needed to confirm its presence irrespective of episodes of use and abstinence so as to ensure there is actually a personality disorder [28].

The study was approved by the Ethical Committee of Clinical Research of Galicia (Spain) (2010–011). All clinical investigation has been conducted according to the principles expressed in the Declaration of Helsinki. Participants provided their written informed consent in all cases. All potential participants who declined to participate or otherwise did not participate were eligible for treatment (if applicable) and were not disadvantaged in any other way by not participating in the study.Research was not conducted outside of our country of residence. No current external funding sources for this study.

### Statistical Analysis

Statistical analysis was performed using Statistical package for the social sciences for Windows-SPSS (version 15). For the comparison of qualitative variables the chi-square test was used. The level of statistical significance was set at p<0.05 for intergroup comparisons with the required post-hoc corrections when comparisons where made with the chi-square.

Multiple correspondence analysis was used for exploratory analysis in order to identify association trends between the explanatory variables and the results.

## Results

### Substance Abuse

The main substances of abuse reported in our sample were: alcohol (42.5%), opiates (35%), cocaine (13%), cannabis (2.9%), and other substances (2.2%). In 101 patients (4.3%) more than one main substance was registered as none met the three, previously defined requirements [26], [27]; in this group of patients the most frequent combination was the use of opiates and cocaine in 41 patients (40.6% of this group). Sociodemographic and substance use variables are shown in table 1.

**Table 1:** Sociodemographic characteristics and main substance of abuse.

Sociodemographic characteristics (N = 2300)	Mean	T. D.
Age	41.27	10.13
	n	%
Sex		
Male	1834	79.7
Female	466	20.3
Main substance of abuse Harmful abuse/dependence)		
Opiates	805	35
Cocaine	300	13
Alcohol	978	42.5
Other	217	9.4

The different groups created as a function of the main substance of abuse for statistical analysis were: the OP group (opiates), the COC group (cocaine), the ALC group (alcohol) and the OS group (other substances: cannabis, nicotine, psychoactive drugs or more than one substance).

Taking into account the heterogeneity as regards the main substance as well as the reduced size of the group OS, this group was excluded for later intergroup comparisons.

63.9% of the sample was polydrug users. Polydrug use frequency for each main substance was as follows: ALC (49.4%), OP (79.1%) and COC (70%). In the ALC group the main substances found in polydrug use were: cocaine (8.7% of total, 17.6% of polydrug use), cannabis (7.36% of total, 14.9% of polydrug use) and opiates (1.02% of total, 2.07% of polydrug use); in the OP group the main substances used in polydrug use were: cocaine (43.1% of total, 54.4% of polydrug use), cannabis (30.7% of total, 38.7% of polydrug use) and alcohol (19.2% of total, 24.3% of polydrug use); Finally, in the COC group the main substances in polydrug use were: Alcohol (41% of total, 52.11% of polydrug use), cannabis (35% of total, 44.5% of polydrug use) and opiates (13.3% of total, 17% of polydrug use).

### Mental and Behavioral Disorders

Co-occurrence between mental disorders and SUD reached 56.3% of the total sample and 14.1% of patients showed more than one mental disorder besides the addictive disorder.

The most prevalent diagnostic categories in the sample were mood disorders (22.3%), personality disorders (20.5%) and anxiety disorders (14.3%). The full list of diagnostic categories and of mental disorders diagnosed is shown in Tables 2, 3 and 4.

**Table 2:** Prevalence of mental disorders; cognitive, psychotic and mood disorders.

	n	%
Some diagnosis of mental disorder	1298	56.3
More than one diagnosis of a mental disorder	323	14.1
Some diagnosis of Axis I mental disorder	969	42.1
Some diagnosis of Axis II mental disorder	465	20.2
Delirium, dementia and amnesic and other cognitive disorders	6	0.3
Delirium	2	0.1
Dementia	4	0.2
Schizophrenia and other psychotic disorders	169	7.3
Schizophrenia	48	2.1
Schizophreniform disorder	7	0.3
Schizoaffective disorder	7	0.3
Delusional disorder	19	0.8
Brief psychotic disorder	9	0.4
Substance-induced psychotic disorder	66	2.9
Psychotic disorder not otherwise specified	13	0.6
Mood disorders	511	22.2
Major depressive disorder	93	4
Dysthymic disorder	153	6.7
Manic episode	2	0.1
Hypomanic episode	4	0.2
Bipolar disorder	37	1.6
Cyclothymic disorder	14	0.6
Substance-induced mood disorder	141	6.1
Mood disorder not otherwise specified	67	2.9

**Table 3:** Prevalence of anxiety, somatoform, dissociative, eating and impulse-control disorders.

	n	%
Anxiety disorders	330	14.3
Panic disorder	38	1.7
Agoraphobia	2	0.1
Specific phobia	1	0.05
Social phobia	10	0.4
Obsessive-compulsive disorder	15	0.7
Posttraumatic stress disorder	11	0.5
Acute stress disorder	6	0,3
Generalized anxiety disorder	64	2,8
Anxiety disorder due to general medical condition	12	0.5
Substance-induced anxiety disorder	123	5.3
Anxiety disorder not otherwise specified	48	2.1
Somatoform disorders	20	0.9
Somatization disorder	1	0.05
Conversion disorder	3	0.1
Pain disorder	5	0.2
Hypochondriasis	9	0.4
Undifferentiated somatoform disorder	2	0.1
Dissociative disorders	3	0.1
Dissociative amnesia	1	0.05
Dissociative amnesia disorder	2	0.1
Eating disorders	26	1,1
Anorexia nervosa	5	0.2
Bulimia nervosa	8	0.3
Eating disorder not otherwise specified	13	0.6
Impulse-control disorders not otherwise specified	117	5.1
Intermittent Explosive disorders	19	0.8
Kleptomania	2	0.1
Pathological gambling	28	1.2
Impulse-control disorders not otherwise specified	68	3

**Table 4:** Prevalence of adjustment and personality disorders.

	n	%
Adjustment disorder	39	1,7
Personality disorder	465	20.2
Paranoid personality disorder	31	1.3
Schizoid personality disorder	17	0.7
Schizotypal personality disorder	9	0.4
Histrionic personality disorder	33	1.4
Borderline personality disorder	119	5.2
Antisocial personality disorder	106	4.6
Narcissistic personality disorder	11	0.5
Obsessive-Compulsive personality disorder	6	0.3
Avoidant personality disorder	7	0.3
Dependent personality disorder	26	1.1
Personality disorder not otherwise specified	100	4.3

### Axis I Diagnoses

42.5% of the sample had Axis I disorders with significantly lower presence in the OP group in relation to the other two groups (Table 5).

**Table 5:** Diagnostic categories and most frequent diagnoses per groups.

	1. OpiatesN = 805 n (%)	2. CocaineN = 300 n (%)	3. AlcoholN = 978 n (%)	x^2^	Significant intergroupdifferences
Delirium, dementia, amnestic and other cognitive disorders	–	1(0.3)	5(0.5)	4.04	
Schizophrenia and other psychotic disorders	61(7.6)	38(12.7)	46(4.7)	23.24***	1–2, 1–3, 2–3
Schizophrenia	23(2.9)	6(2)	11(1.1)	7.04*	1–3, 2–3
Substance- induced disorder	22(2.7)	21(7)	18(1.8)	21.67***	1–2, 2–3
Mood disorders	166(20.6)	55(18.3)	235(24)	5.59	
Major depressive disorder	25(3.1)	13(4.3)	49(5)	4.02	
Dysthymic disorder	59(7.3)	15(5)	64(6.5)	1.93	
Substance-induced mood disorder	35(4.3)	10(3.3)	84(8.6)	18.60***	1–3, 2–3
Anxiety disorder	96(11.9)	53(17.7)	147(15)	6.92***	1–2, 1–3
Generalized anxiety disorder	17(2.1)	18(6)	24(2.5)	12.96**	1–2, 2–3
Substance-induced anxiety disorder	32(4)	19(6.3)	62(6.3)	5.37	
Somatoform disorders	10(1.2)	3(1)	5(0.5)	2.93	
Eating disorders	4(0.5)	8(2.7)	8(0.8)	11.21**	1–2, 1–3, 2–3
Impulse control disorders not elsewhere classified	24(3)	23(7.7)	43(4.4)	11.63**	1–2, 2–3
Impulse control disorders not otherwise specified	18(2.2)	16(5.3)	19(1.9)	1114**	1–2, 2–3
Personality disorder	213(26.5)	50(16.7)	155(15.8)	35.27***	1–2, 1–3
Borderline personality disorder	57(7.1)	21(7)	32(3.3)	14.87***	1–3, 2–3
Antisocial personality disorder	74(9.2)	6(2)	16(1.6)	62.76***	1–2, 1–3
Personality disorder not otherwise specified	22(2.7)	11(3.7)	49(5)	6.12*	1–3

* p<.05,

** p<.01,

*** p<.001.

The schizophrenia and psychotic disorders group (7.3%) manifested mainly in the COC group, with significant differences in relation to the OP and ALC groups. Of these, the substance-induced psychotic disorder (2.9%) was significantly more frequent in the COC group than in the other groups while schizophrenia (2.1%) was significantly higher in the COC and OP groups in comparison to the ALC group.

As to mood disorders, the main diagnoses were dysthymic disorder (6.6%), substance-induced mood disorder (6.2%) and major depressive disorder (4%), with no differences among groups except for the substance-induced mood disorder, which was significantly more frequent in the ALC group.

Among anxiety disorders (14.3%), substance induced anxiety disorder (5.3%) and generalized anxiety disorder (2.8%) were the most frequent, with a significantly higher presence of this latter disorder in the COC group.

Somatoform, dissociative and eating disorders (ED) affected a small proportion of patients (0.8, 0.1 and 1.1% respectively). The latter were significantly more frequent in the COC group.

Impulse-control disorders (ICD) not elsewhere classified (4.8%) were significantly more frequent in cocaine users than in the remaining groups, which was also true of ICD-not otherwise specified.

Lastly, only 1.2% of patients in the sample displayed an adjustment disorder.

The distribution of the more frequent different diagnostic and mental disorder categories relative to the different groups of SUD is displayed in table 5.

### Axis II Diagnoses

20.2% of patients were diagnosed with some personality disorder (PD). Such disorders were more frequent in the OP group.

Particularly salient were Borderline Personality Disorder (BPD: 5.2%), Antisocial Personality Disorder (APD: 4.6%) and the personality disorder not otherwise specified (4.3%). At the other end of the scale, obsessive compulsive personality disorder (0.3%) and avoidant personality disorder (0.3%) had the fewest diagnoses.

BPD was significantly more frequent in the OP and COC groups in comparison to the ALC group while APD was strongly associated to the OP group and personality disorder not otherwise specified was associated to the ALC group.

**Figure 1:**
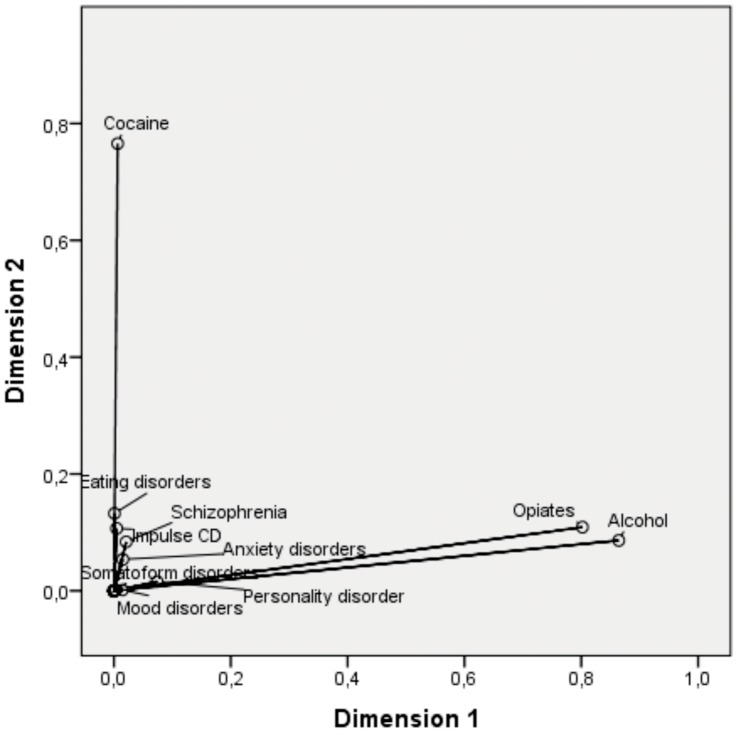
Multiple correspondence analysis of mental disorders and substance abuse.

The multiple correspondence analysis performed found that substance of abuse groups are distributed separately into: COC group, and OP and ALC groups (figure 1). Equally remarkable was spatial distribution as cocaine abuse is at one extreme while opiate and alcohol abuse is at the other extreme. As shown in figure 1, the mental and behavioral disorders are all distributed together and all of them very separated from substance abuse groups. This is explained by the fact that only part of drug users suffer from a mental and behavioral disorder and use is non-related. Closeness of diagnostic categories to one use or the other is in line with the prevalence each substance has as seen in the descriptive analysis.

## Discussion

The prevalence of mental disorders in the COPSIAD study (56.3%) is close to that obtained in the *Epidemiologic Catchment Area-*ECA study (60%) [4], and significantly lower than that of the *National Comorbidity Survey-* NCS (78.3%) [29] and very similar to that found in the NCS-R study (55%) [5], [6], all of them baseline studies in the field (although there were conducted in non-clinical populations using different methodology).

The distribution for the most frequent mental disorders obtained was similar to that reported in the pilot study by Szerman *et al.* [8] in Madrid (Spain), although the prevalence of dual disorder was significantly lower in the latter study, which also included patients from the mental health network who tend to present with dual disorder less frequently than patients seen in the addictive disorders network.

A case in point is Rush & Koegl (2008) [11], where prevalence of dual disorder varies depending on where within the mental health system patients are assessed: 28% in hospitalized patients, 19.1% in intensive outpatient programs and 17.8% in regular outpatient treatment.

A common finding in many studies is the close association between cocaine use and the presence of mental disorders [4], [7], [8], [29].

In our study, the presence of Axis I mental disorders was significantly lower in the OP group than in the COC and ALC groups. In all three groups there was a predominance of mood disorders, which coincides with the NCS [29] and was also largely consistent with percentages in the ECA groups, with the exception of alcohol users who were more frequently diagnosed with anxiety disorders [4]; in the NCS-R anxiety disorders were also more frequent [5], [6].

Mood disorders (22.3%) were more frequent in the ALC group (24%), with a significant association between substance-induced mood disorder and alcohol use. The association between affective disorders and alcohol use was been reported in many previous studies: as many as 45% of patients with a diagnosis of alcohol dependency satisfy the criteria of major depression. Nonetheless, after 4 weeks of abstinence, the percentage decreases to 6%. On the other hand, alcohol-abstinent patients are four times more likely to develop a depressive disorder. Furthermore, the efficacy of the antidepressant treatment is decreased if there is alcohol use involved [30].

It might be that the serotonergic depletion caused by chronic alcohol abuse accounts for these findings [30]. The low level of polydrug use of the ACL patients in our study confirms this special association between depression and chronic alcohol abuse.

Anxiety disorders were also frequent, with a significant association between these disorders and the COC group. The increase in noradrenergic activity at the locus coeruleus, typical of stimulating agents such as cocaine would worsen anxiety symptoms [30].

Like other authors [8], we found a predominance of psychotic disorders in the COC group, particularly relevant in substance-induced psychotic disorders, which seems reasonable as psychosis is one of the most common complications of cocaine use.

It is estimated that up to 68% of cocaine addicts display abuse-related psychotic manifestations [31]. Cocaine’s ability to block up to 77% of dopamine reuptake would account for the appearance of these disorders [30]. These psychotic symptoms may persist for months after cocaine use has stopped or even become chronic.

Another diagnostic category with an unexpectedly high prevalence was impulse control disorders (ICD) not elsewhere classified. Their frequency was significantly higher in the COC group. This seems only reasonable as this substance may alter the inhibiting ability of the prefrontal cortex and increase the limbic emotional response which may lead to the manifestation of impulsive behaviors [30].

On the other hand, the relatively high presence of ICDs not otherwise specified (3%) coupled with the inexistence of a substance-induced ICD diagnosis in the DSM IV-TR, may lead us to think that the impulsivity “trait” and the impulsivity “state” are being intermingled, notably in the COC group (5.3%).

Eating disorders (ED) were significantly more frequent in the COC group. In this regard, it was been found that the greater the severity of the ED, the higher is the number of the substances abused [32], which may account for the results obtained although their low prevalence (1.1%) precludes drawing any conclusion on the matter.

On the other hand, the comorbidity obtained in our study in Axis II (20.5%) is quite below that reported by other authors [33], [34]. This may be due to a variety of causes such as:

- the use of psychometric instruments in some studies [33], [34], which could possibly increase the number of Axis II diagnoses. Indeed, in some studies a single individual is diagnosed with several different personality disorders [33], [34], which can hardly be accepted from a clinical point of view.
- time of assessment: our study only includes patients with a follow-up of at least 3 months so as to maximally reduce the number of patients who might be under the effect of substance intoxication or withdrawal, which might contribute to explain the psychopathological symptoms attributable to these contexts as an part of an independent mental disorder.

Contrary to what we saw in the case of Axis I, we find a higher comorbidity for Axis II disorders for all groups of non-alcoholic substances (OP: 26.5%, COC; 16.7%) than in the alcohol group (ALC: 15.8%), although the difference is only significant for the OP group. This had already been reported in earlier studies such as NESARC [35].

The most frequent personality disorders (PD) were borderline- BPD (5.3%) – considered as the most prevalent personality disorder among substance abusers [36], and the antisocial-APD (4.6%) and PD not otherwise specified (4.3%). The high prevalence of BPD and APD reported among substance abusers may be associated to impulsivity (common to both disorders) whose role as vulnerability factor for addiction development has been widely acknowledged [37].

These results are similar to those reported by Barea *et al*. [33], although this latter study yields higher prevalences (BPD: 17.4%; PD not otherwise specified: 14.3%; APD: 12.6%), probably because of the type of population targeted in the study (mainly opiates users) and the method of diagnosis (IPDE- International Personality Disorder Examination).

Much higher prevalences were found in the ATOS study [34], conducted on a sample of 615 heroin users, where 71% of patients were diagnosed with APD, 46% with BPD and 38% satisfied the criteria for both disorders.

We believe that the lower prevalence of PD found in our study may be attributable to the preeminent role given to clinical diagnosis.

Obviously, conclusions are applicable to the outpatient population seen at addictive disorders units, and may not be generalized to the universe of substance abusers. In this regard, the seriousness of dual disorder increases as we get closer to specialized services, among other things because patients with more than one psychiatric disorder are more likely to demand specialized assistance [38], [39].

Graphical representation of correspondence analysis indicates that there are two major groups of users: cocaine users on the one hand and opiates and alcohol on the other. The mental and behavior disorders are not clearly associated to a specific substance abuse group. All mental and behavior disorders are grouped close together but far from substance abuse groups. The reason for this is that in this sample the diagnosis of substance abuse is central for sample collection. Particularly remarkable is the fact that in the results obtained opiates and alcohol groups go together while the cocaine group stands alone. This would indicate different patterns in substance abuse as shown clearly in the descriptive analysis.

In summary, our study indicates the presence of a high prevalence of mental disorders in a clinical population of patients seen in addictive disorders units. High polydrug use, particularly in the case of the OP and COC groups, together with a high presence of substance-induced Axis I disorders, and whose association to use groups is clearly physiopathologically based (depression to alcohol, psychosis and impulsivity to cocaine) seem to confirm the absence of a clear tendency towards the abuse of a given substance on the basis of a previous psychopathology [40].

As to Axis II disorders, the high use of cocaine in patients from the OP group does not allow us to state that impulsivity and emotional instability predispose to a greater use of opiates. Furthermore, there are no significant difference in the BPD between the OP and the COC groups and it is highly likely that the higher number of criminal behaviors associated to the abuse of inhaled and injected substances increases the diagnosis of antisocial PD [40] in the OP group.

The main strengths of this study are its sample size and the use of DSM IV-TR diagnostic criteria that allow for discrimination between “primary disorders” and “substance-induced disorders” [28].

A potential limitation of this study is the absence of structured interviews to diagnose mental disorders in this type of populations such as the Psychiatric Research Interview for Substance and Mental Disorders (PRISM) [41], although we are of the opinion that the fact that longitudinal diagnoses are made by the patient’s clinicians is an added value.

Other limitations were: the inclusion of cases in each unit, which was not uniformly done, and might have had implications on the sample collection procedure; no data on the evolution of toxic substance use was collected and no temporal sequencing was established regarding the onset of disorders.

Bearing all these factors in mind, we believe that the important sample size (n = 2300) provides us with valuable information on the reality found in the addictive disorders units of Galicia.
